# Advances in Enzyme-Based Biosensors for Pesticide Detection

**DOI:** 10.3390/bios8020027

**Published:** 2018-03-22

**Authors:** Bogdan Bucur, Florentina-Daniela Munteanu, Jean-Louis Marty, Alina Vasilescu

**Affiliations:** 1 National Institute of Research and Development for Biological Sciences, Centre of Bioanalysis, 296 Splaiul Independentei, 060031 Bucharest, Romania; bucurica@yahoo.com; 2 Faculty of Food Engineering, Tourism and Environmental Protection, “Aurel Vlaicu” University of Arad, Elena Dragoi, No. 2, 310330 Arad, Romania; florentina.munteanu@uav.ro; 3 BAE Laboratory, Université de Perpignan via Domitia, 52 Avenue Paul Alduy, 66860 Perpignan, France; 4 International Centre of Biodynamics, 1B Intrarea Portocalelor, 060101 Bucharest, Romania; avasilescu@biodyn.ro

**Keywords:** pesticide, enzyme inhibition, biosensor

## Abstract

The intensive use of toxic and remanent pesticides in agriculture has prompted research into novel performant, yet cost-effective and fast analytical tools to control the pesticide residue levels in the environment and food. In this context, biosensors based on enzyme inhibition have been proposed as adequate analytical devices with the added advantage of using the toxicity of pesticides for detection purposes, being more “biologically relevant” than standard chromatographic methods. This review proposes an overview of recent advances in the development of biosensors exploiting the inhibition of cholinesterases, photosynthetic system II, alkaline phosphatase, cytochrome P450A1, peroxidase, tyrosinase, laccase, urease, and aldehyde dehydrogenase. While various strategies have been employed to detect pesticides from different classes (organophosphates, carbamates, dithiocarbamates, triazines, phenylureas, diazines, or phenols), the number of practical applications and the variety of environmental and food samples tested remains limited. Recent advances focus on enhancing the sensitivity and selectivity by using nanomaterials in the sensor assembly and novel mutant enzymes in array-type sensor formats in combination with chemometric methods for data analysis. The progress in the development of solar cells enriched the possibilities for efficient wiring of photosynthetic enzymes on different surfaces, opening new avenues for development of biosensors for photosynthesis-inhibiting herbicides.

## 1. Introduction

Nowadays, with the aim of achieving high productivity in agriculture, pest control is managed through the use of a wide variety of intentionally toxic compounds, pesticides which are released into the environment with serious consequences. The total amount of pesticides used in 2015 was about 3.42 × 10^6^ t/y worldwide, of which 0.36 × 10^6^ t/y was in Europe (2015 data from FAOSTAT (FAO, 2018)). The justification for use of these pesticides relies on the assurance of food and feed quantity and quality. Unfortunately, even when used according to the regulations, only a minor amount of pesticides reaches the proposed targets while the rest represents environmental contaminants, with serious negative impact as some compounds are persistent in the environment, their half-lives being of several decades [1]. Directive 2009/128/EC concentrates on the achievement of a sustainable use of pesticides in the EU through the reduction of risk and impact on human health and the environment. Meanwhile, it also promotes the use of non-chemical alternatives to pesticides. 

**Figure 1:**
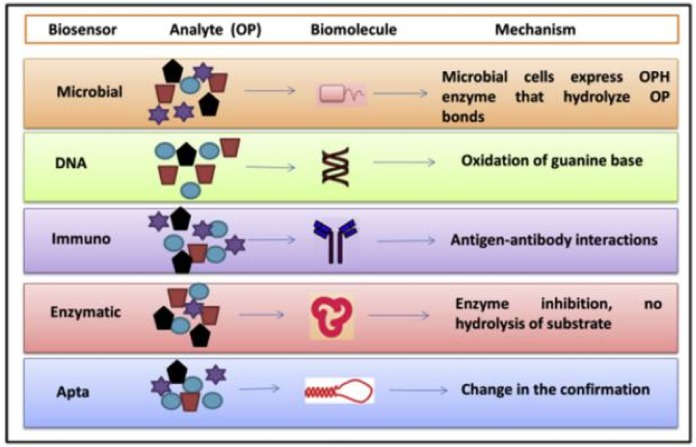
Reprinted from [12] with permission from Elsevier. Principle of biosensors based on different biomolecules aimed at organophosphorus compounds (OP) detection.

The standard analytical methods that are used for the detection of pesticides are chromatographic techniques coupled with various detectors [2,3]. These methods have the advantages of being automated and accurate, with high specificity and can be used for simultaneous detection. However, these systems are suffering from some drawbacks, such as high costs, being time consuming, the need for sample pre-treatment, a slow response time, and the requirement for skilled personnel. Therefore, the research focused on finding fast and reliable devices, biosensors, which can be used for pesticide detection [4,5,6,7,8,9,10,11,12] (Figure 1).

Various types of biorecognition elements can be used in biosensors to achieve specific and sensitive recognition of target pesticide in complex mixtures: whole cells or subcellular fragments of microorganisms, enzymes, antibodies, DNA sequences, aptamers, or enzymes. Since many pesticides have been designed on the principle of inhibiting a key enzymatic process, the biosensors based on enzymatic inhibition are particularly relevant as they perform quantitative detection of a certain pollutant based on its toxicity. This review focuses on this type of biosensor and aims to present its advances and current limitations.

## 2. Detection of Neurotoxic Insecticides Based on Cholinesterase Inhibition

The use of insecticides in agriculture is strictly controlled by complex and contradictory regulations set to each individual compound. For example, in the EU the organophosphorus compounds (OP) are either approved (chlorpyrifos, dimethoate, malathion) or banned (parathion, coumaphos diazinon). A similar situation exists for carbamate compounds (CB) with both approved (pirimicarb, methomyl, formetanate) or banned (carbofuran, carbaryl, aldicarb) examples [13]. There are also complex rules and risk evaluations for each insecticide (including the corresponding metabolites/degradation compounds). For example, in the case of chlorpyrifos-methyl the highest risk was determined by the European Food Safety Authority (EFSA) for foods, with wheat and rye grain being the main contributors; maximum residue levels were set for different foods in the 0.01−2 mg/kg range [14]. There is an important need for fast and sensitive analytical methods for screening a large number of food and environmental samples contaminated with minute quantities of different insecticides/metabolites in complex matrices. The toxicity mechanism of the neurotoxic insecticides is based on the inhibition of acetylcholinesterase (AChE; EC 3.1.1.7) [15], and the reproduction of this inhibition in vitro can be used for multianalyte selective monitoring. There are reported to be numerous biosensors developed for the detection of neurotoxic organophosphorus and carbamate insecticides based on the inhibition of cholinesterase that were reviewed concerning their general aspects [16], the parameters influencing the enzymatic inhibition such as the effect of substrate concentration, enzyme total activity or the presence of organic solvents [17], strategies for biosensor construction using various immobilization methods and the roles of various matrices used [18], improvement of the selectivity and sensitivity using genetically engineered mutant enzymes [19], biosensor integration in flow analytical manifolds [20,21], use of special detection techniques such as piezoelectric quartz crystal microbalance [22], combination with various kinds of nanomaterials [23], impact of cutting-edge technologies [24], specific application for fast screening of food samples [25], etc. Most biosensors were based on electrochemical detection [26], while optical [27,28,29,30] and piezoelectric detection [22] were more rarely explored. The vast majority of published AChE-based biosensors are able to provide only an indication of the toxic compounds present in samples—expressed as a global inhibition percentage caused by all the insecticides and other interfering compounds—that is useful only to identify the suspicious samples that must be further investigated by complex chromatographic techniques. In this section, we review special developments in the field of cholinesterase-based biosensors for neurotoxic insecticide analysis that try to address the above-mentioned disadvantage using various strategies such as the development of artificial neural networks, the combination of AChE with other enzymes, or specialising a sample pre-treatment in order to obtain more reliable information. 

## 3. Use of Chemometric Methods for Enhancement of AChE Biosensors Performances

The combination of analytical responses from various biosensors can be mathematically interpolated using techniques such as partial least squares (PLS), artificial neural networks (ANNs), or multiple curve resolution methods in order to discriminate between different samples/analytes and reveal information that is not directly available using only one detection technique [31]. In the case of biosensors based on AChE the use of chemometric techniques is based on two facts: (i) the inhibition of AChE produced by insecticide mixtures is higher than the individual inhibition percentages produced by each individual inhibitor [32] and (ii) the enzymes extracted from various organisms or genetically engineered have variable sensitivities for different insecticides [19].

Chemometrics methods, such as principal component regression (PCR), partial least squares (PLS), and radial basis function-artificial neural network (RBF-ANN) were compared and tested for the simultaneous detection of carbaryl and phoxim in mixtures by spectrometric measurements, using 5,5′-dithiobis(2-nitrobenzoic) acid (DTNB) as a chromogenic reagent. The best performing model was based on the RBF-ANN method with good recovery of the pesticides from spiked water samples [33].

One of the first examples of ANNs for discrimination of neurotoxic insecticides using AChE biosensors is the simultaneous detection and resolution between paraoxon (an OP) and carbofuran (a CB), using the signals obtained with four different biosensors, each one based on a different AChE extracted from electric eel, bovine erythrocytes, rat brain, and *Drosophila melanogaster* [34]. The artificial neural network (ANN) was developed by feeding the algorithm with the inhibition percentages measured with each biosensor for various mixtures of paraoxon and carbofuran with different concentrations. The system was able to discriminate between paraoxon and carbofuran in mixtures with a concentration of 0–20 μg L^−1^ for each analyte, with prediction errors of 0.9 μg L^−1^ for paraoxon and 1.4 μg L^−1^ for carbofuran [34]. The proposed system was subsequently improved by using genetically engineered variants of *Drosophila melanogaster* AChE wild-type and mutants Y408F, F368L, and F368H, each variant being selected due to individual sensitivity patterns towards paraoxon and carbofuran, respectively. In order to achieve an even higher resolution, a variant with extremely diminished paraoxon sensitivity F368W was added into the network. The use of engineered enzymes allowed the analysis of binary paraoxon and carbofuran mixtures with concentrations 0–5 μg L^−1^, with prediction errors of 0.4 μg L^−1^ for paraoxon and 0.5 μg L^−1^ for carbofuran. Interestingly, the system was also able to be adapted for the discrimination of two OP insecticides; malaoxon and paraoxon mixtures with concentrations 0–5 μg L^−1^ were discriminated with prediction errors of 0.9 and 1.6 μg L^−1^ [35], and thus the possibility of investigating even insecticide mixtures from the same class was demonstrated. 

**Figure 2:**
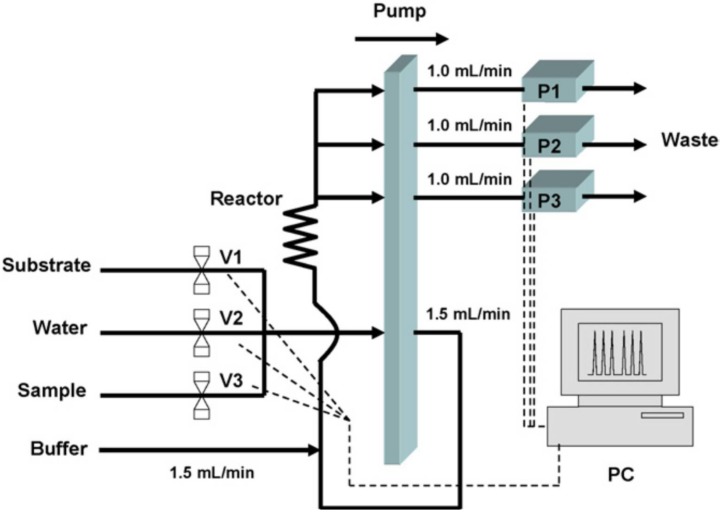
Scheme of the Flow Analysis system. V1–V3: pinch valves that select only one solution that will be aspirated by the peristaltic pump; P1–P3: potentiostats each one allocated to a biosensor inserted in a flow cell. Reprinted from [36] with permission from Elsevier.

After the proof of concept demonstration of insecticide discrimination using ANNs with different AChE variants, the subsequent work aims to address the main disadvantages: the relative important number of enzymes variants required and the reduction of the analysis time. In order to decrease the analysis time, an automated flow analysis manifold was used to measure the same sample simultaneously, with three different biosensors based on AChE from Electric eel and two different genetically modified enzymes from *Drosophila melanogaster*, in order to discriminate paraoxon and dichlorvos (see Figure 2). Thus, the analysis time with the proposed system is similar to the one based on a single enzyme (mono-channel) and also the reproducibility is improved by the reduction of the human errors. 

Another improvement in comparison with previous proof of concept is also the reduction of the number of enzyme variants from four to three, using enzymes with important relative variations between the inhibition constants k_i_ and ANNs trained by employing back-propagation algorithms with 17 training solutions accordingly to a full factorial design and another set of 8 solutions generated randomly inside the training space used to evaluate the model’s predictive ability [36]. 

**Figure 3:**
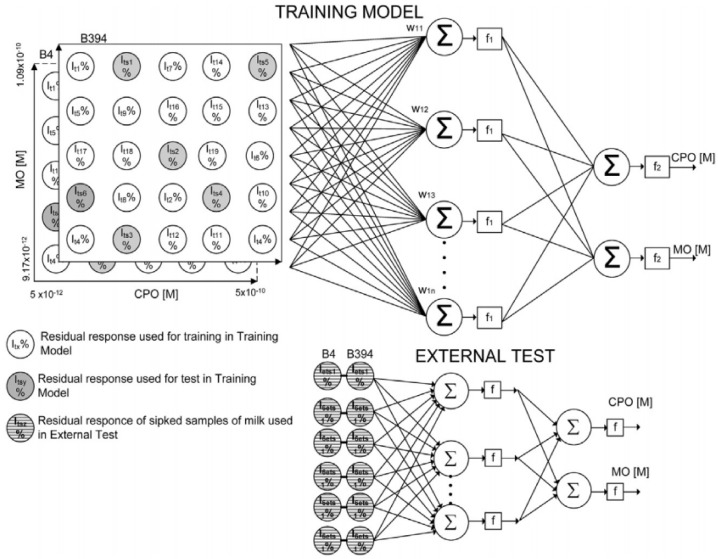
The artificial neural network (ANN) constructed based on a set of data divided in 19 training set and 6 tests set. Reprinted from [37] with permission from Elsevier.

Subsequent development was the further reduction of enzymes required for the discrimination of chlorpyriphos-oxon and malaoxon to only two genetically engineered variants: B4 and B394, a mutant with a higher sensitivity, both from *Drosophila melanogaster*. The biosensors were inserted in a flow cell and used in a flow system in order to have a short analysis time and good reproducibility. A set of 19 solutions with various concentrations of both insecticides was used for the development of the ANN, and the modelling was validated with another six solutions of mixtures of insecticides with known concentrations. The ANN was built using a combination of 9 training algorithms and 14 different structures with a different number of neurons, hidden layers, and different transfer functions (logsig–logsig, logsig–purelin, tansig–tansig–purelin). The final selected parameters included an input layer with 2 neurons, a hidden layer with 10 neurons, and an output layer with 2 neurons (Figure 3). The range and concentrations for each insecticide in the mixture were chosen from the inhibition curves obtained with standard solutions that contain a single insecticide and were 1.09 × 10^−10^ to 5.0 × 10^−12^ M for chlorpyriphos-oxon and 1.01 × 10^−9^ to 9.17 × 10^−11^ M for malaoxon in milk samples [37]. 

**Figure 4:**
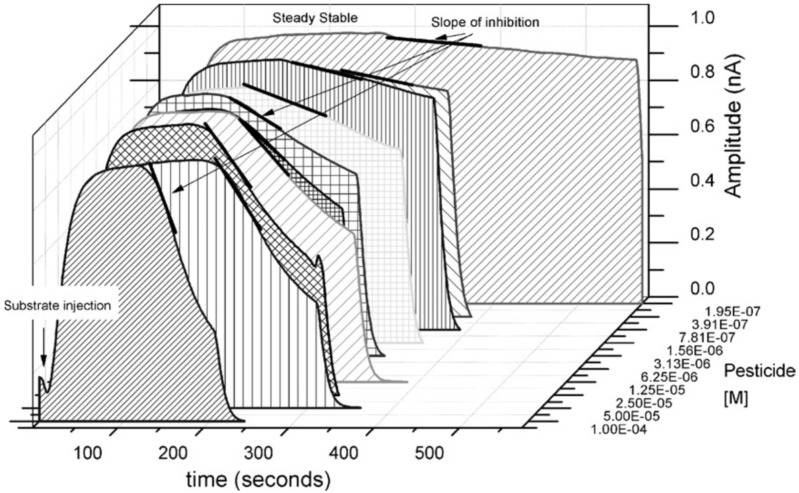
Measurement principle and the analytical signals used for measurement of enzyme inhibition rate. Reprinted from [38] with permission from Elsevier.

A further extension of ANN performances was the composite solving of a mixture with three insecticides (chlorpyriphos-oxon, chlorfenvinphos, and azinphos-methyl-oxon) using only two AChEs from *Drosophila melanogaster* (wild-type and B394, genetically modified) that have differential sensitivity and specificity toward organophosphorus insecticides. The feat was achieved based on a combination of two factors: (i) development of an ANN with three biosensors obtained with each separate enzyme and a combination of both enzymes and (ii) the use of a more complex analytical signal (enzymatic activity rate) instead of the inhibition percentage. The biosensors with enzyme immobilization on the electrode surface are not designed for the calculation of all the constant rates involved in an enzyme-catalyzed reaction by direct equations (enzymes being used free in the solution for these studies). The authors have determined an apparently irreversible inhibition constant by measuring in batch the slope of the current decrease due to the inhibition (current/time) from the steady-state signal corresponding to the initial enzymatic activity (see Figure 4). The concentrations of insecticide were determined by the inhibition measured for each analyte separately and the relative toxicity of each analyte: from 0.1 nM to 1 µM for chlorfenvinphos (the weakest inhibitor), and ranged from 0.1 nM to 0.1 µM for both azinphos-methyl-oxon and chlorpyriphos-oxon. The ANN model was developed using two subsets of standard solutions with insecticide mixture: 43 mixed solutions for the training subset and 20 mixed solutions for the test. The final selected parameters of the ANN included an input layer with three neurons, a hidden layer with 95 neurons, and an output layer with 3 neurons [38].

ANNs have proven their performances and possibility to extend to numerous insecticides, but suffer from several drawbacks: they are relatively complicated, the presence of an unknown insecticide in the sample may be difficult to identify, or they are unsuitable for real samples that contain a large number of toxins. Another system based on two different AChEs intends to improve the original purpose of the biosensors: to serve only as an alarm for the identification of the presence of insecticides in complex real samples in the presence of interferences, and not to discriminate between various compounds. The demonstrative system was constructed using two biosensors: (i) based on the E69W mutant AChE from *Drosophila melanogaster* sensitive to omethoate and (ii) an omethoate-resistant AChE from electric eel. The same sample was analyzed with two biosensors based on two enzymes: if only the E69W mutant was inhibited then the omethoate was present, while an inhibition of both enzymes was an indication of other interfering compounds such as heavy metals or sodium hypochlorite [39].

**Figure 5:**
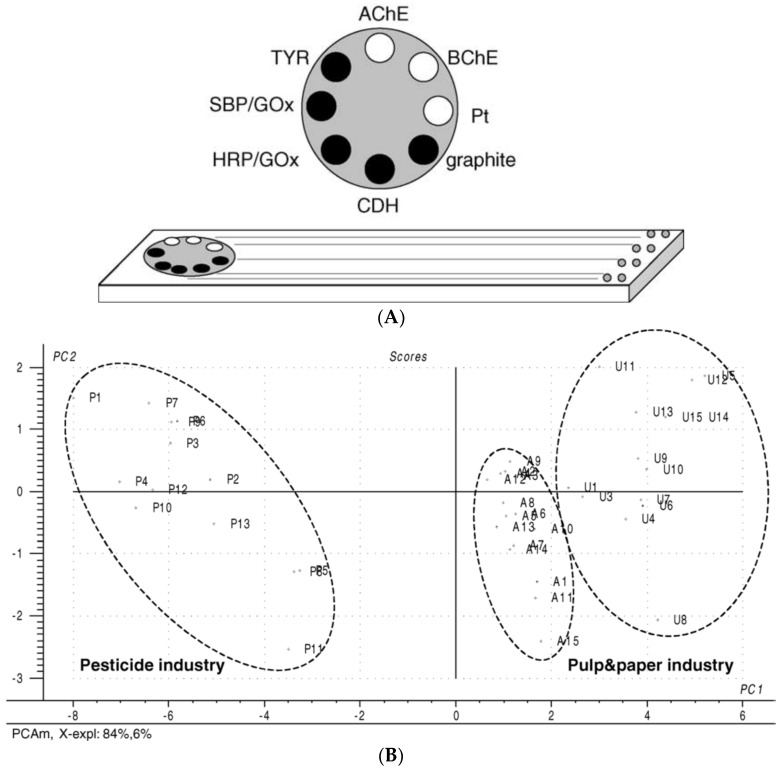
(**A**) The construction of the eight-electrode screen-printed array; black: graphite and white: platinum electrodes free or modified with enzymes; (**B**) PCA score plot for the first two principal components obtained from environmental samples. P represents pesticide samples whereas A and U samples represent two subsets of paper wastewater varying in toxicity content. Reprinted from [41] with permission from Elsevier.

Besides ANN, another interesting chemometric technique that allows the characterization and segregation of complex samples using data from various analytical methods is the principal component analysis (PCA). The goal of the PCA is to classify an unknown sample in a specific group based on data obtained from several semi-selective analytical techniques, the samples being represented in a graph where they are being spatially differentiated in groups based on complex mathematical algorithms [40]. One such example of a multi-sensor is based on eight different electrodes: AChE biosensor, butyryl cholinesterase, tyrosinase, glucose oxidase mixed with either horseradish peroxidase or soybean peroxidase, cellobiose dehydrogenase, and uncoated Pt and graphite electrodes (see Figure 5A). The measurements were carried out at different potentials depending on the enzymatic substrate/product used for each enzyme-electrode couple, for example, 350 mV for cholinesterase modified and bare platinum electrodes; 400 mV for cellobiose dehydrogenase modified and bare graphite electrodes; or −100 mV for tyrosinase, horseradish, and soybean peroxidase modified electrodes. Model standard solutions were used containing phenols (phenol, catechol, p-aminophenol, p-chlorophenol and p-cresol) and pesticides (heptenophos, dichlorvos, carbaryl, fenitrothion and phosphamide) in order to discriminate between wastewater samples. Thirteen samples from the pesticide industry and 30 samples from a pulp and paper industry (at two different concentration levels) were used. Following data treatment (including pretreatment to avoid drift) a clear separation was obtained between the different sample groups in the representation of score-loading bi-plot PC1 and PC2 of the data variance (see Figure 5B) [41]. 

## 4. (Bio)chemical Sample Treatment in Combination with AChE Biosensors

One of the main hurdles preventing biosensors application in real-life applications is their susceptibility to provide false signals in the case of samples with an unknown complex matrix that may contain interferents. One possibility to address this issue is to carry out some sample pretreatments in order to remove or mitigate some interferences. One obvious treatment is the addition of buffers that, besides pH regulation, may also lead to the precipitation of some interferents (such as heavy metals). Another more complicated system is the analyte selective preconcentration by solid phase extraction using columns filled with XAD 2 sorbent. The analytes were eluted from the column using water miscible organic solvents that raised other problems due to unspecific interactions with the enzymes. The authors found the 5% acetonitrile to be an optimum working condition for measurement with biosensors in a batch system and the columns also allowed the heterogeneous oxidation of parathion to paraoxon to further improve the limits of detection due to the increase in inhibition rate [42].

Besides the inhibition in water-miscible organic solvents, there are reports that demonstrate the possibility of using hydrophobic solvents for pesticide extraction from the sample and inhibition. The initial and remnant enzymatic activity is measured in aqueous medium (phosphate buffer) and only the inhibition step is made in organic solvent, that is, 10 mL of isooctane used for simple sample extraction by mixing and centrifugation. The analyte extraction in isooctane allows avoidance of the nonspecific enzyme denaturation produced by sample matrix (pH or other nontoxic components from fruit juices) and also recovers the activity of the inhibited enzyme treatment with 2-PAM [43]. Another non-miscible organic solvent used is hexane, which allows maintenance of 100% of the enzyme activity and has the supplementary advantage of avoiding inhibition by interferences produced by heavy metals [44]. There are reports on the use of hydrophobic organic solvents in mixtures such as chloroform-n-hexane (50%, *v*/*v*) for detection of aldicarb or paraoxon [45], and as a general rule the screen-printed electrodes must be avoided in the development of biosensors operating in organic media because the organic solvents may damage the inks or the plastic substrate. 

Phosphotriesterase is an enzyme with a very similar structure to the binuclear nickel center in urease without a known naturally occurring substrate, but which has the ability to detoxify organophosphate insecticides and military chemical substances from the same class [46]. Besides the main detoxification use, the phosphotriesterase was also used for the enhancement of the AChE-based biosensors. Chlorfenvinfos (on organophosphate insecticide) is a substrate that acts as competitive inhibitor of phosphotriesterase and prevents the efficient hydrolysis of other pesticides such as chlorpyrifos or paraoxon. Based on this particular property, a network formed from an AChE biosensor and a bienzymatic biosensors based on AChE and phosphotriesterase were used for the identification of chlorfenvinfos presence in insecticide mixtures using chlorpyrifos as example. Thus, if the AChE biosensor is inhibited and AChE coupled with phosphotriesterase presents no inhibition then an organophosphate insecticide other than chlorfenvinfos is present in the sample, while if both the AChE biosensor and AChE coupled with phosphotriesterase are inhibited then the sample contains chlorfenvinfos [47]. In a spectrophotometric system, phosphotriesterase in combination with two types of AChEs from electric eel and the recombinant B394-from *Drosophila melanogaster* were also used for the discrimination of the carbamate presence in of chlorpyriphos-oxon and dichlorvos organophosphate insecticides, based on the class detoxification of phosphotriesterase and the use of an ANN with a single hidden layer containing four neurons [48].

Another enzyme that raised interest in detoxification or prophylactic and post-exposure treatments for organophosphate insecticides or nerve agents is organophosphate hydrolase [49]. Organophosphate hydrolase can be used for the biosensor development to directly detect organophosphate insecticides based on their enzymatic degradation [50] or coupled with AChE for improved selectivity. One such example is the discrimination of paraoxon (an organophosphate compound) in the presence of carbofuran (a carbamate insecticide), based on the difference between the non-additive AChE inhibition produced by both insecticides in a mixture (measured without organophosphate hydrolase) and the inhibition produced only by paraoxon (measured in the presence of organophosphate hydrolase). Thus, the biosensor is able to indicate the type of inhibitor [51].

## 5. Detection of Photosynthesis-Inhibiting Herbicides

Herbicides are widely used in agriculture for weed control based on different modes of action. While the lists of approved herbicides and their maximum residue levels allowed in food, feed, and water in different regions of the world are constantly revised, some herbicides such as atrazine, which has been banned in the European Union since 2000, are still approved to be used in other regions of the world, for example, atrazine continues to be used in North America and Asia. Current methods for herbicides detection rely on gas or liquid chromatography procedures coupled with mass spectrometry detection, capillary electrophoresis, and ELISA [52]. Herbicides belonging to phenylurea (e.g., diuron, linuron etc.), triazine (atrazine, simazine cyanazine etc.), diazine (bromacil, lenacil etc.), and phenol chemical groups (dinoseb, ioxynil, bromoxynil etc.) inhibit photosynthesis in plants, cyanobacteria, algae, and diatoms. Several biosensors for the detection of photosynthesis-inhibiting herbicides have exploited the very mode of action of these pesticides [52]. In photosynthesis, plants, cyanobacteria, and diatoms convert light into energy, the first step in this process being to split water and produce oxygen and protons at the end of an electron transfer chain. This happens in Photosystem II (PSII, water-plastoquinone oxidoreductase), a protein complex located in the thylakoid membrane of plants, algae, and cyanobacteria. Herbicides bind at the level of protein D1 in PSII, blocking the electron transfer and inhibiting photosynthesis. According to their chemical structure, herbicides bind to different amino acids in protein D1.

The activity of PSII is not only inhibited by certain herbicides but also by endocrine disruptive compounds [53], heavy metals [54], explosives like TNT [55], or ionizing radiation [56]. 

The sensitivity to herbicides depends not only on the specific interaction between a particular herbicide and the photosynthetic enzymes but also on the type of photosynthetic element and its preparation. Isolated PSII systems proved to be more sensitive than thylakoid membranes or whole cells, as cells possess, in addition to the cell membrane acting as a diffusion barrier, protective intracellular mechanisms that help preventing to a certain extent the effect of herbicides. 

Without providing an exhaustive list of biosensors developed so far for which details can be found in several reviews [52,57], Table 1 summarizes some of the most representative examples of biosensors based on photosynthetic elements for the detection of herbicides. These include optical biosensors based on measuring changes in chlorophyll fluorescence and electrochemical ones, based either on quantitative measurement of the oxygen produced via a classic Clark electrode or by using an artificial mediator such as dicholoroindophenol (DCPIP). Another possibility is to measure the rate of photoreduction by the PSII of an artificial mediator (e.g., duroquinone, 2,5-dichlorobenzoquinone etc.). Immobilization of photosynthetic enzymes was achieved by physical adsorption, electrostatic interactions (e.g., in layer-by-layer procedures), entrapment in polymers (alginate, agar, polyvinyl alcohol bearing styryl pyridinium groups—PVA-SbQ etc.), or cross-linking with BSA–glutaraldehyde [52].

As the data in Table 1 emphasize, the biosensors based on photosynthetic enzymes have a low selectivity and while some PSII-based biosensors can detect phenylurea and phenolic herbicides at the ppb level [52], in accordance with current European Regulations regarding maximum pesticide residue levels in water, others are much less sensitive. Moreover, the number of practical applications for the analysis of surface and ground waters remains very limited to this day. Their performances recommend these biosensors as a screening tool and indicator of “total toxicity”. They are helpful for a first evaluation of a high number of environmental samples to identify the “toxic” samples that should be analyzed in detail by costly analytical methods like GC/MS or LC/MS.

A decade ago, in a 2008 review, Campas et al. [57] emphasized the efforts made by researchers towards advancing photosynthesis-based biosensors to meet the sensitivity and selectivity requirements for a successful commercially viable bioanalytical tool addressing current regulations on herbicides. More specifically, efforts were directed to combining sample pre-concentration with biosensor analysis, or to using photosynthetic systems isolated from mutant strains to increase sensitivity and selectivity. For example, solid phase extraction using cyanazine molecularly imprinted polymer (MIP) cartridges was coupled with analysis with a biosensor that used thylakoid membranes from spinach and fluorescence detection [20,77]. As an alternative approach, multi-biosensors were developed which employ several photosynthetic preparations from wild-type and different mutant strains with different sensitivity to herbicides [78]. This strategy allowed discrimination of triazines from phenylurea and triazinine herbicides, and the improvement of sensitivity to match the limits set by European regulations [79].

These efforts continued in the last decade searching for new mutants with enhanced sensitivity or resistance to herbicides to be included in portable instruments [67,71]. To widen the purpose of analysis and to address the detection of multiple analytes, photosynthetic biosensors were coupled with other enzyme biosensors. Moreover, electrochemical and optical modules have been included on the same detection platform, for example, in the multielectrode array for analyzing sugars, phenols, and pesticides reported by Scognamiglio et al. [71]. Researchers investigated new immobilization methods (e.g., laser-induced forward transfer-LIFT [70], layer-by-layer [62]) for the purpose of achieving fast, reproducible modification of sensing interfaces with photosynthetic bioelements in a nondestructive manner. Important advances made in this direction in recent years have originated from works devoted to developing hybrid devices able to perform artificial photosynthesis. Efficient wiring of photosynthetic enzymes to electrode surface was accomplished, for example via conductive polymers [80].

Various photosynthetic elements of different purity and size, from pure PSII preparations to thylakoid membranes, PSII-enriched thylakoid membrane fractions (“BBY particles”), and up to whole cells have been studied to obtain the biorecognition element with the best sensitivity, selectivity, and stability attributes (Table 1). The stability of photosynthetic preparations is quite low generally, particularly for pure PSII preparations—a few hours at room temperature [68]. Thylakoids from spinach deposited by LIFT have a half-life of 1 day at room temperature but can be stored for more than 3 months at −20 °C without losing their activity [70], while *Chlamydomonas reinhardtii* whole cells immobilized by physical adsorption on silicon septum were stable for 1 month at room temperature [63].

Several attempts to increase the stability of photosynthetic preparations have been reported that focused on either adding catalase to remove reactive oxygen species formed upon illumination of thylakoids [65], or on using PSII complexes from thermophilic bacteria [69], in addition to new immobilization procedures. 

**Figure 6:**
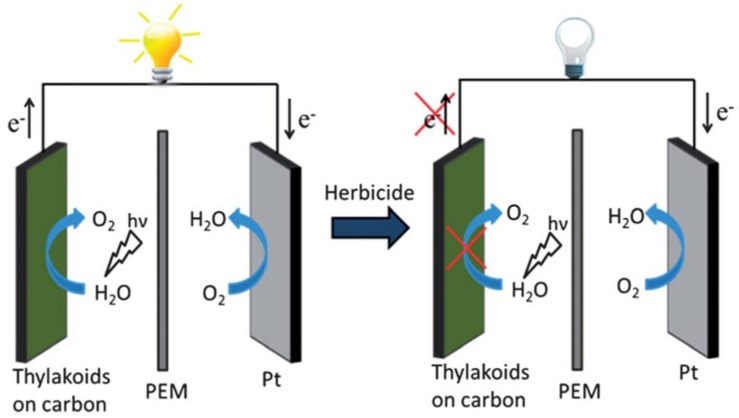
Schematic of the self-powered herbicide biosensors showing the light powered bioanode before (left) and after (right) inhibition of thylakoid membrane bio-solar cell by the addition of herbicide. Reproduced from [65] with permission from the Royal Society of Chemistry.

Illustrative examples of the progress of photosynthesis-based biosensors towards real-life applications include a self-powered biosensor and a multi-biosensor herbicide analyzer. The self-powered biosensor for detection of herbicides in water was designed as a solar cell, with thylakoid membranes at bioanode and with a Pt cathode. Photosynthesis-inhibiting herbicides decreased the current output of the biosolar cell (Figure 6). The limits of detection for commercial herbicides atrazine, bromacil, and diuron were below 0.5 μg L^−1^, (below the EPA limits) and the linear response spanned up to ~15 μg L^−1^ [65].

A 4-channel flow-through system including electrodes modified with photosynthetic thylakoid membranes from *Spinacia oleracea* L., *Senecio vulgaris*, and its mutant resistant to atrazine was used for detecting multiple herbicides, based on the different sensitivity of the four PSII receptors to herbicides. The detection limit achieved with this biosensor was 10^−8^ M in river water in spring [61].

**Figure 7:**
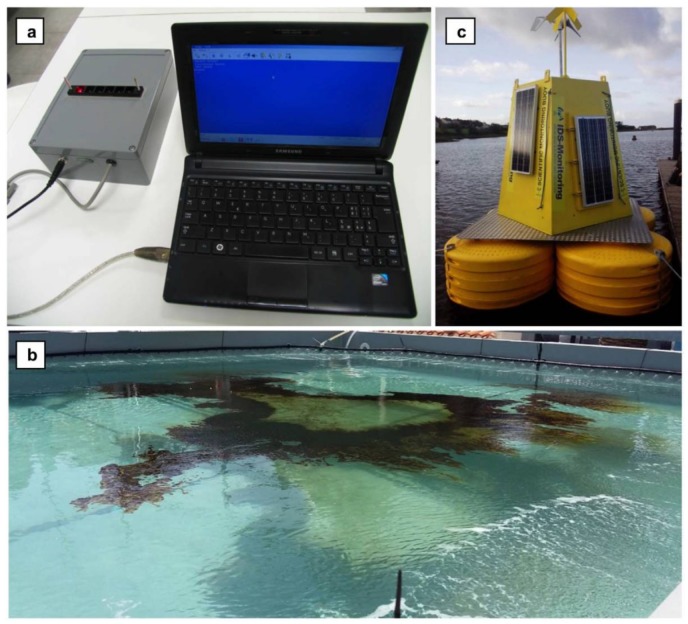
(**a**) Six-cells portable fluorimeter developed by Biosensor Srl (Italy) for analysis of microalgae fluorescence responses in the presence of seawater pollutants. (**b**) Seawater oil spill simulation in an outdoor mesocosm tank at the Institute for Coastal Marine Environment (Messina, Italy); the perimeter tubing system to simulate the pelagic environment and prevent hydrocarbon adhesion is observable. (**c**) Automated telemetry-operated marine buoy (IDS Monitoring Ltd., Clare, Ireland) where the microalgae bioassay and fluorescence instrumentation for pesticide screening were installed. Reprinted from [75] with permission from Elsevier.

There was notable progress in the development of portable instruments for herbicide analysis, in particular with fluorescence-based detection. Commercial detection kits for herbicides based on Chlamydomonas (with amperometric or fluorescence detection) or on thylakoids (with amperometric detection) are available from Biosensor SRL in Italy (http://www.biosensor-srl.eu/). Additionally, multi-cell devices incorporating various photosynthetic biorecognition elements are increasingly tested with real samples and in in field conditions [61,75]. Most recently, real-time, autonomous screening of pesticides in coastal areas was achieved with a bioassay based on a device incorporated into a marine buoy, that combined green microalgae and fluorescence detection [75]. (Figure 7).

Inkjet-printing of viable photosynthetic cyanobacteria, retaining their photosynthetic activity on carbon-nanotube modified paper was demonstrated [81], while intense research is conducted in the field of biosolar cells and self-powered biosensors [65], particularly focusing on the efficient wiring of photosynthetic enzymes to different supports [80]. This stands as proof of the interest in photosynthetic enzymes and the huge potential of combining all these new ideas towards herbicide detection. 

## 6. Other Enzymes

### 6.1. Alkaline Phosphatase

Alkaline phosphatase (ALP) known also as basic phosphatase has a broad substrate specificity and exhibits maximum activity in alkaline pH solutions. ALP is a metalloenzyme that has in its active center Mg^2+^ and Zn^2+^ ions, a reason for which is inhibited by a series of heavy metals, organophosphorus pesticides, and inorganic salts. Its characteristic of catalyzing the reaction of numerous inorganic and organic compounds makes this enzyme interesting for use in the construction of biosensors for toxicity screening. 

García Sánchez et al. [9], were using a sol–gel modified with ALP for construction of a biosensor for the screening of heavy metals and pesticides. In their experiments, a fluorimetric ALP-based biosensor was used for the detection of different inhibitors of the enzymatic activity. The biosensor was obtained through the microencapsulation of ALP in sol–gel matrices derived from tetramethyl orthosilicate.

The constructed system was able to detect organochlorine (tetradifon), carbamate (metham-sodium), and organophosphorus pesticides (fenitrothion) and some inorganic compounds. The linear ranges and the detection limits for the screened pesticides are reported in Table 2. 

The inhibition results obtained with free and immobilized ALP showed that the ALP biosensor can be used for the pollutants screening, and the detected pesticides were reported as model compounds for non-specific inhibition of a range pollutants.

**Figure 8:**
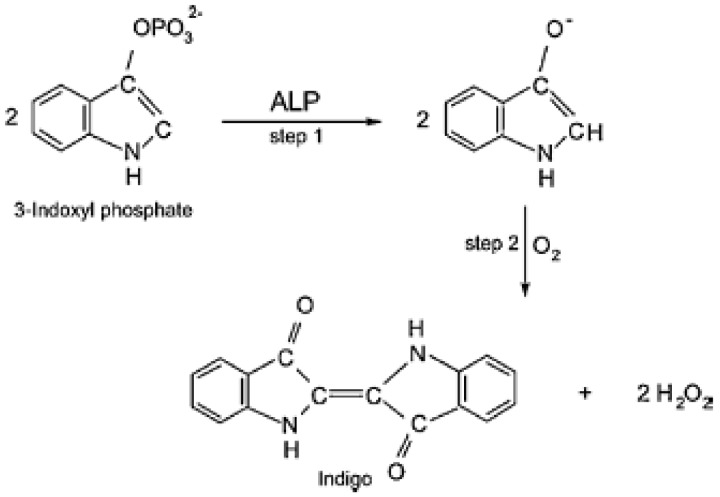
Reaction sequence at the electrode: step 1 is catalyzed by ALP, while step 2 is the spontaneous oxidation of the enzymatic reaction product. Reprinted from [90] with permission from the Royal Society of Chemistry.

Mazzei et al. [90] proposed an inhibition–amperometric biosensor that is based on the reactions pictured in Figure 8.

For the construction of the biosensor, ALP was immobilized on a nylon 6,6 membrane that has carboxylic groups on its surface and then was attached to an amperometric hydrogen peroxide sensor, which was monitoring the changes in the hydrogen peroxide concentrations that are proportional to the concentration of 3-indoxyl phosphate (3-IP). In the presence of a pesticide, ALP is inhibited and the concentration of 3-IP decreases, which leads to a decreased signal for the detected hydrogen peroxide. The biosensor proposed by Mazzei et al. [90] has the advantage of not needing a regeneration of the electrode due to the fact that the inhibition process is reversible. Unfortunately, the biosensor suffers from lack of specificity.

In the paper published by Ayyagari et al. [5], the ALP catalyzes the dephosphorylation of a macrocyclic compound with the release of light that is detected by a simple photomultiplier tube. ALP was immobilized through the building of a molecular assembly of the enzyme and a conjugated copolymer, poly(3-undecylthiophene-co-3-thiopbnecarboxyldehyde-biotin-LC-hydrozone) on a glass surface. For this purpose, the immobilization of the biotinylated copolymer on a silanized glass surface was performed using the hydrophobic or specific biotin–streptavidin interactions. Afterwards, a streptavidin conjugate of alkaline phosphatase was attached to the copolymer. The decrease of the chemiluminescent signal strength is directly proportional to the inhibition of the enzyme activity by the presence of paraoxon. The presented biosensor has the advantage that is reusable a number of times without a significant loss of enzyme activity.

**Figure 9:**
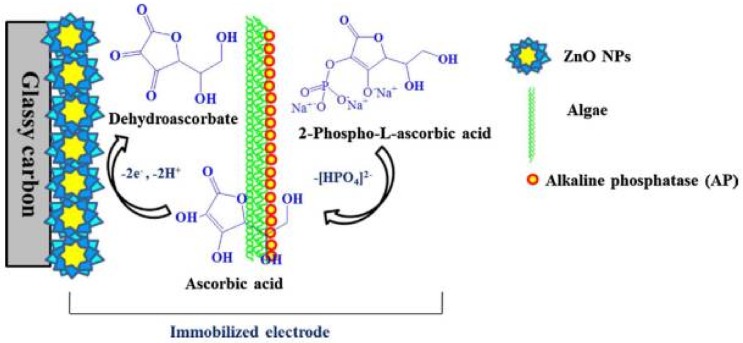
Mechanism of reaction occurring at AP-algae/ZnO/GC electrode. Reprinted from [91] with permission from Elsevier.

A voltammetric detection of another pesticide, chlorpyrifos, was performed using a biosensor modified with ALP [91]. The detection principle of this biosensor was based on the inhibition of AP-algae in presence of chlorpyrifos. For the construction of the biosensor a glassy carbon electrode was used, onto which were placed ZnO nanoparticles that had the role of increasing the conductivity and/or to increase the electron transfer rate between the electrode surface and the immobilized algae. Algae-BSA was deposited on the newly obtained electrode surface and allowed to react for 15 min, and thereafter, the electrode was immersed in glutaraldehyde to crosslink the amines provided by BSA (Figure 9). 

The obtained biosensor was used for selective voltammetric detection of chlorpyrifos without interferences from malathion, acephate, triazophos, and alkali metals [91].

### 6.2. Organophosphorus Hydrolase

Organophosphorus hydrolase (OPH; E.C. 3.1.8.1), first isolated from *Pseudomonas diminuta* [92] is a well-characterized metalloenzyme that has the ability to hydrolyze a large variety of organophosphate pesticides [93], and the resulting hydrolysis products change the pH of the solution. The change of the solution pH is due to the generation of two protons during the organophosphate hydrolysis, which takes place with the cleavage of the P-X bonds.

Simonian et al. [94] prepared an optical biosensor by using gold nanoparticles modified with reactive *sulfo*-*N*-hydroxy succinimide to which OPH was covalently bound through the lysine residues. This biosensor detection was based on the relationship variation of the fluorescence with the change in distance between nanogold nanoparticle and fluorophore. 

**Figure 10:**
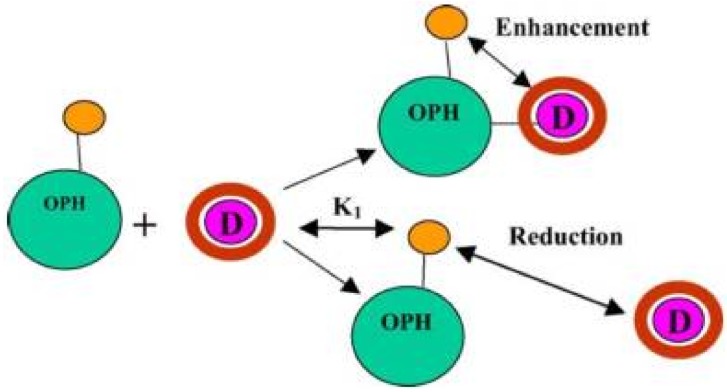
Schematic of Decoy-Enzyme interaction for enhancement in the absence of substrate. Decoy (D) binds to enzyme–nanogold conjugate (OPH), leading to a surface enhanced fluorescence of the decoy. Reproduced from [94] with permission from Elsevier.

In their approach, the OPH-gold nanoparticle conjugates were incubated either with a fluorescent enzyme inhibitor or with a decoy (Figure 10).

The obtained biosensor was used for detection of paraoxon, and the best sensitivity was achieved when the decoy and OPH-gold nanoparticles were present in the system in equimolar levels. An important advantage of this biosensor was that the pH measurement was excluded from the experimental work, which made the assay much easier [94].

### 6.3. Tyrosinase

Tyrosinase enzymes (EC 1.14.18.1, monophenol, *o*-diphenol: oxygen oxidoreductase) are found in many species of bacteria and are copper-containing, dioxygen activated enzymes, that catalyze the transformation of monophenolic and diphenolic compounds as specific substrates to *o*-diphenols and quinones, respectively. Tyrosinase is involved in melanin formation and in the enzymatic browning of fruits. Tyrosinase is inhibited by various environmental pollutants, including hydrazines, atrazine, cyanide, and diethyldithiocarbamate pesticides. Several electrochemical biosensors have been developed for pesticides using tyrosine as biorecognition element, for example, the device proposed by Kim et al. [95], designed with a view to preserve tyrosinase activity after immobilization. This was achieved through the use of reduced pyrroloquinoline quinone (PQQ) covalently bound to gold nanoparticles deposited onto a glassy carbon electrode. The biosensor was used to detect 2,4-dichlorophenoxyacetic acid by amperometry. The presence of the PQQ contributed to reversibility of the system and to an improved sensitivity and simplicity. [95] 

**Figure 11:**
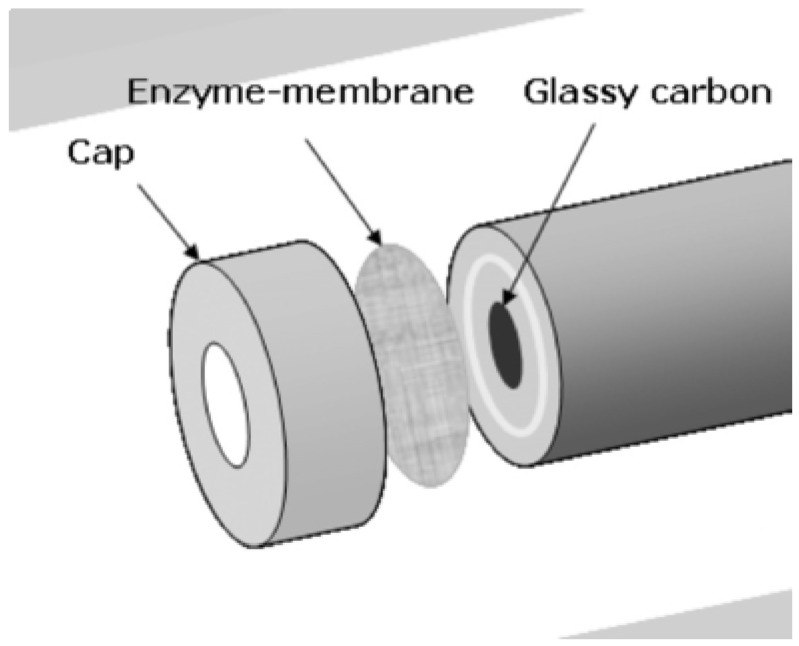
The assembly of the prepared enzyme-membrane with a glassy carbon electrode for pesticide biosensor. Reprinted from [96] with permission from Elsevier.

Shim et al. [96] filled the pores of a bromomethylated poly (2,6-dimethyl-1,4-phenylene oxide) (BPPO) single-layered membrane with cross-linked polyvinyl alcohol (PVA) containing tyrosinase. The modified membrane was attached to a glassy carbon electrode (Figure 11). 

In their paper, the authors compared the results obtained with the tyrosinase in the *p*BPPO membrane and showed that their biosensor had superior performance to other biosensors used for parathion and carbaryl [96,97,98,99]. Moreover, this biosensor showed long-term operational stability, due to the good stability of the tyrosinase modified membrane. 

Exceptional sensitivity with a 0.1 ppt detection limit was reported for atrazine detection with an amperometric biosensor relying on tyrosinase-immobilized vertical growth TiO_2_ nanotubes (Tyr/TiO_2_−NTs). [82] The sensor was applied for the detection of atrazine in soil samples and the results were similar to those obtained by a standard chromatographic method [82].

An interesting biosensor for detection of atrazine was prepared by using a composite obtained through the polymerization of L-DOPA in presence of tyrosinase and thereafter immobilized on a gold electrode in the presence of Nafion. The amperometric detection of atrazine was based on its inhibition effect on tyrosinase catalytic activity and the biosensor used proved good stability, high sensitivity, and precision [84].

Tortolini et al. [83] studied the role of the immobilisation procedure and the type of electrode on the sensitivity of amperometric biosensors for atrazine. Catechol was added as substrate to measure the activity of tyrosinase. Multi-walled carbon nanotubes electrodes, modified with tyrosinase by enzyme entrapment in polyvinyl alcohol bearing styrylpyridinium groups (PVA-SbQ), performed better than graphene or graphite electrodes with the enzyme immobilised with Nafion membrane or by covalent binding in a bovine serum-glutaraldehyde film. Along with a detection limit of 0.3 ppm, the method had a good accuracy as indicated by recovery factors close to 95% in spiked drinking water samples [83].

### 6.4. Laccase

Laccase (polyphenol oxidase, EC 1.10.3.2) belongs to the group of copper-containing oxidases and catalyzes the oxidation of various organic compounds such as phenols in the presence of molecular oxygen. While laccase was widely used in biosensors for the detection of phenolic compounds [100], its application in inhibition-based sensors for pesticides was more rarely pursued.

Zapp et al. [101] developed a biosensor for the detection of methomyl (C_5_H_10_N_2_O_2_S), an insecticide belonging to the carbamate pesticides, which is used for protection of agricultural crops [101]. For the construction of the biosensor, laccase (*Aspergillus oryzae*) was immobilized on platinum nanoparticles dispersed in 1-butyl-3-methylimidazolium tetrafluoroborate as ionic liquid prepared in montmorillonite and then mixed with graphite powder and assembled in a 1 mL syringe. This biosensor is also based on the inhibition of the enzyme by the pesticide, in presence of dopamine as a substrate for laccase. 

The results obtained with this biosensor for determination of methomyl from carrots and tomatoes are similar to the ones obtained through a chromatographic method [101].

Oliveira et al. [102] concentrated on a number of carbamate pesticides that were determined using laccase from *Trametes versicolor.* This laccase was directly immobilized on Prussian blue functionalized carbon paste doped with graphene [102]. The presence of the Prussian blue film had the role of reducing the charge transfer resistance and the biosensor capacitance. The quantification of the carbamates of interest (see Table 2) was based on the inhibition of laccase by using 4-aminophenol as substrate. 

The main advantage of this biosensor resides in the fact that the laccase was directly immobilized on the Prussian blue film in acidic conditions without any cross-linking agents. In another study by this group [103], laccase (*Trametes versicolor*) was immobilized on multiwalled carbon nanotubes and used to obtained carbon paste electrodes. By means of square wave voltammetry the inhibition effect of pirimicarb on the laccase activity was monitored in the presence of 4-aminophenol as enzyme substrate. The optimum results for the design of this biosensor were attained with a composition of composite carbon paste consisting of 60:40% (*w*/*w*) MWCNTs and paraffin binder in which the laccase was dispersed. 

Detection of a carbamate pesticide, formethanate hydrochloride, could be achieved with the help of an electrochemical biosensor based on laccase. Laccase was cross-linked to a gold electrode onto which gold nanoparticles were electrodeposited. The working mechanism of the biosensor is based on the formethanate hydrochloride inhibition of the laccase catalytic reaction that takes place in the presence of phenolic compounds. The laccase-based biosensor which was developed provided a good performance for monitoring formethanate from real samples, grapes and mango [85].

### 6.5. Heme-Containing Enzymes

House fly cytochrome P4506A1 was confined in dioctadecyl dimethyl ammonium bromide (DDAB) film. This mimicking of a bio-membrane was attached to an edge plane pyrolytic graphite electrode and was used for the amperometric detection of two organochlorine pesticides, aldrin and heptachlor. The results that were obtained confirmed the fact that the main pathway for the oxidation of the studied pesticides by the cytochrome P4506A1 was the epoxidation, and that this biosensor is prone to the detection of aldrin and heptachlor [104].

An amperometric biosensor based on horseradish peroxidase (HRP) was used for the detection of dichlofenthion, by monitoring the formation of 2,4-dichlorophenol from the hydrolysis reaction of the pesticide with OPH. The biosensor was prepared through the overnight adsorption of HRP on the glassy carbon electrode. The dual enzyme electrochemical assay which was developed allows detection of a broad range of organophosphorus pesticides that are not common substrates for OPH [88].

As the list of approved pesticides is constantly evolving, among the pesticides tested as potential analytical targets for urease, special attention was paid to glyphosate (*N*-(phosphonomethyl)glycine). Its popularity is due to the fact that it is a broad-spectrum, non-selective herbicide, that, unfortunately, was used in excessive amounts for the majority of crops just before harvest [105], currently being the world’s most extensively used weed killer [106]. In the literature numerous methods for the detection of this pesticide are described, including biosensors based on the principle of peroxidase inhibition [86,87].

Peroxidases catalyze the oxidation of various hydrogen donor compounds in the presence of peroxides. In the work of Oliveira et al. [86] peroxidase isolated from the atemoya fruit was immobilized in montmorrilonite clay at the surface of a carbon paste electrode modified with carbon nanotubes. The enzymatic activity and its inhibition by glyphosate was followed in the presence of enzyme substrate hydroquinone via Square Wave Voltammetry. The limit of detection of the herbicide was 30 µg L^−1^ and the sensor’s accuracy was proven by the good recovery values in spiked water samples. Montmorrilonite provided a favorable medium for urease, contributing to the preservation of enzyme’s stability and activity for 8 weeks [86]. In another approach, horseradish peroxidase (HRP) was immobilized by electrostatic attachment to a nanocomposite polymeric film of poly (2,5-dimethoxyaniline) doped with poly (4-styrenesulfonic acid) (PDMA-PSS) at the surface of a gold electrode [87]. In this configuration, the detection limit for glyphosate was 0.16 µg L^−1^, much lower than the previous example and the metabolite of glyphosate, aminomethylphosphonic acid was detected at 1 µg L^−1^ [87]. Besides glyphosate, peroxidase is also inhibited by heavy metals [107], sulfides [108] etc. therefore a more holistic approach looking at all possible inhibitors within a real sample is desirable when developing a novel peroxidase-based biosensor.

### 6.6. Urease

**Figure 12:**
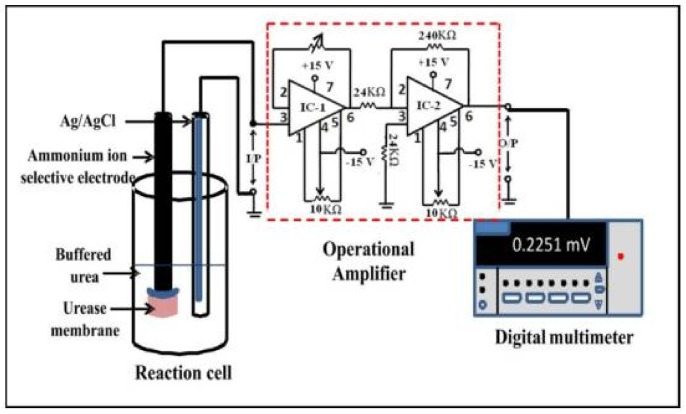
Schematic representation of the experimental set-up for potentiometric measurement with the circuit diagram of the amplifier. Reprinted from [106] with permission from Elsevier.

Urease (EC 3.5.1.5) catalyzes the decomposition of urea in ammonia and carbon dioxide. Its inhibition by heavy metals [109] and pesticides such as atrazine [89] was used as a principle in biosensors for environmental monitoring. The potentiometric biosensor proposed by Vaghela et al. [106] for the detection of glyphosate is based on urease that was immobilized on gold nanoparticles and entrapped in an agarose guar gum membrane. The membrane covered an ammonium ion selective electrode (Figure 12), and the amount of ammonium released in the urease-catalyzed reaction measured with this electrode was directly correlated with enzyme activity in the membrane. A detection limit of 5 × 10^−6^ M glyphosate was reached and the sensitivity and response time were improved compared to the case when urease was entrapped in the membrane in the absence of gold nanoparticles. The enhanced performances were thus attributed to the gold nanoparticles which provided a larger area for urease loading with the preservation of enzyme activity and better conductivity.

This potentiometric biosensor had good selectivity for glyphosate as among the various other pesticides tested (dichlorvos, dimethoate, paraquat dichloride, and hexaconazole) only hexaconazole interfered to some degree. Moreover, the sensor had good reproducibility and a storage stability of 180 days [106].

### 6.7. Aldehyde Dehydrogenase

Aldehyde dehydrogenase (E.C. 1.2.15, AlDH) catalyzes the conversion of aldehydes to carboxylic acids in the presence of the enzymatic cofactor nicotinamide adenine dinucleotide (NAD^+^) or nicotinamide adenine dinucleotide phosphate (NADP^+^), according to the general reaction:

$$\mathrm{Aldehyde}+{\mathrm{NAD}}^{+}\overset{\mathrm{AlDH}}{\to }\mathrm{Acid}+\mathrm{NADH}+H^{+}$$

Following the discovery that dithiocarbamate fungicides inhibit aldehyde dehydrogenase from baker’s yeast, several electrochemical biosensors have been developed by Noguer et al. [110,111,112,113]. Dithiocarbamate fungicides (e.g., ziram, mancozeb, thiram, maneb, zineb, metam sodium etc.) are non-systemic, widely applied fungicides in agriculture to protect fruits and vegetables against fungus. The maximum residue limits for dithiocarbamate fungicides in food are in the range of ppm [114]. Besides dithiocarbamates, benzimidazole fungicides like benomyl also inhibit AlDH. Human exposure to benzimidazole fungicides was linked to the occurrence of Parkinson’s disease [115]. All this underline the importance of fast, sensitive methods for the detection of fungicides inhibiting AlDH.

For analytical purposes, in the AlDH catalyzed reaction shown above, the amount of aldehyde converted into acid can be easily determined quantitatively via the measurement of NADH formed as NADH presents distinct fluorescence, UV absorption, and electrochemical activity compared to NAD^+^. In the presence of dithiocarbamate fungicides the activity of AlDH and the amount of NADH formed decrease, proportionally with the quantity of fungicide. Noguer et al. have developed electrochemical biosensors for the detection of dithiocarbamate fungicides (maneb, zineb, metam-sodium and its metabolite methyl isothiocyanate (MITC)) based on this principle [110,111,113]. The equilibrium of the AlDH-catalyzed reaction is shifted to the reactant side. To favor aldehyde conversion to acid, high concentrations of the cofactor NAD^+^ and alkaline pH values are necessary, the optimum activity for the AlDH from baker’s yeast being observed at pH 9.5–10. Shifting the reaction equilibrium towards the product side along with quantitative determination of the NADH formed can moreover be obtained by coupling the reaction catalyzed by AlDH with a second chemical or enzymatic reaction [110]. For example, NADH was detected with screen-printed electrodes modified with an electrochemical mediator, Meldola Blue. The mediator reacts with NADH at the electrode surface, regenerating the cofactor NAD^+^ and making it available to participate again in the enzymatic reaction:

$$\mathrm{Meldola}\;\mathrm{Blue}\;(\mathrm{ox})+\mathrm{NADH}\to \mathrm{Meldola}\;\mathrm{Blue}\;(\mathrm{red})+{\mathrm{NAD}}^{+}+H^{+}+2e^{-}$$

The reduced mediator is reoxidized electrochemically by amperometry at 0 V vs. Ag/AgCl. The intensity of the electrical current generated by the electrochemical oxidation of NADH is proportional to the amount of aldehyde converted, hence with the enzyme activity. By inhibiting AlDH, the toxic metabolite MITC of the fungicide metam-sodium causes a decrease in the current intensity recorded with the AlDH/Meldola Blue screen-printed biosensor, proportionally correlated with the amount of fungicide. The detection limit for MITC was 100 ppb [110].

An alternative approach proposed by the same group to enhance the sensitivity of the biosensor, is to couple a second enzymatic reaction involving diaphorase or NADH oxidase to reoxidize NADH and regenerate the enzymatic cofactor. Diaphorase reoxidizes NADH in the presence of hexacyanoferrate(III) as electron acceptor and the resulting hexacyanoferrate(II) is electrochemically oxidized at a potential of 250 mV versus SCE (Satured Calomel Electrode) [113]. The bienzymatic biosensor containing both enzymes entrapped in a gel of PVA-SbQ was highly sensitive to maneb with a detection limit of 1.48 ppb, significantly better compared to the 400 ppb limit of the classic spectrophotometric method.

A similar biosensor relying on AlDH and NADH oxidase co-immobilized in PVA-SbQ at the surface of a Pt-sputtered carbon paste screen printed electrode achieved a detection limit of 8 ppb of zineb (reported as the solubilized form of the fungicide with disodium EDTA) [111]. The use of thermophilic NADH oxidase is advantageous over the more labile diaphorase, however the higher price, increased complexity of bienzymatic sensors, and irreversibility of the inhibitory effect of fungicides over AlDH emphasize the advantages of monoenzymatic mediator/AlDH biosensors as disposable devices for fungicide detection.

Besides these few reports from 1997–2001, there are no recent studies regarding biosensors for the detection of dithiocarbamate fungicides in environmental and food applications. However, the interest in aldehyde dehydrogenase’s inhibition by pesticides remains quite high in the biomedical field, due to the link between pesticide exposure and Parkinson’s disease [115]. Advancing knowledge regarding the link between the variations in enzyme structure and the effect of pesticides [115], combined with the discovery of novel enzymes from extremophile microorganisms with increased stability and altered selectivity [116,117], might provide novel opportunities in the future for the development of AlDH-based biosensors.

## 7. Conclusions

The biosensors based on enzymatic inhibition are useful as an alarm or general toxicity indicator for the fast identification of the samples contaminated with pesticides. Nonetheless, when multiple mutant enzymes with different characteristics are used in multi-biosensor devices combined with chemometric methods for data analysis, they are also able to provide more complex information concerning the inhibitor, discrimination from mixtures, or elimination of potential interferences. Development of miniaturized, multi-biosensors and the use of nanomaterials continues to draw much research effort, continuing the trends indicated in previous reviews dedicated to biosensors for pesticides. The goal is to reduce the gap between standard methods and accelerate the path towards commercial implementation. However, the number of applications involving real environmental or food samples and their variety remains limited and toxic metabolites of pesticides have rarely been studied. Development of novel biosensors relying on enzymes such as aldehyde dehydrogenase or heme-containing enzymes appears to have stagnated and lost interest in the last decade, probably for reasons including the unavailability of commercial enzymes, difficulties related to price, cofactor addition, or unfavorable equilibrium of the enzymatic reaction or low selectivity. At the same time, novel enzymes are continuously researched as analytical targets for the detection of inhibiting pesticides in assays that may be easily adapted to biosensor formats, for example, hydroxyphenylpyruvate dioxygenase inhibition, recently employed in a biosensor for β-triketone herbicides [118].

The progress in the development of solar cells enriched the possibilities for efficient wiring of photosynthetic enzymes on different surfaces, opening new avenues for the development of biosensors for photosynthesis-inhibiting herbicides. Many different types of nanomaterial-modified interfaces are currently available commercially that can be used to develop biosensors, and screen-printed electrodes are especially attractive for developing disposable electrochemical biosensors for pesticides. While the data presented in the review highlight the major focus on enhancing sensitivity and stability, less attention was devoted to improving the stability of enzymes, which continues to be a bottleneck in the further development of enzyme-based biosensors. Solutions might come from studies of enzymes from extremophiles, genetically modified enzymes, novel surface nanostructuring strategies, and from the interaction between enzymes and nanomaterials, intensively researched as a means to achieve higher biocatalyst stability.

## Figures and Tables

**Table 1:** Examples of biosensors based on the inhibition of photosynthetic enzymes.

Pesticide/Real Sample	Photosynthetic Enzyme	Detector	Analytical Performance ^1^	Reference
Diuron	PSII particles from *Synechococcus bigranulatus*	Amperometry	I_50_ = 9 × 10^−9^ M LOD = 7 × 10^−10^ M	[58]
Diuron Atrazine Simazine Ioxynil Bromoxynil Dinoseb	PSII particles from *Synechococcus elongatus*	Amperometry	Diuron: LOD: 5 × 10^−10^ M, I_50_: 8 × 10^−8^ M Atrazine: LOD:2 × 10^−9^ M, I_50_: 3 × 10^−7^ M Simazine: LOD: 1 × 10^−8^ M, I_50_: 8 × 10^−7^ M Ioxynil: LOD: 9 × 10^−9^ M, I_50_: 4 × 10^−7^ M Bromoxynil: LOD: 2 × 10^−7^ M, I_50_: 8 × 10^−6^ M Dinoseb: LOD: 6 × 10^−8^ M, I_50_: 8 × 10^−7^ M	[59]
Diuron Atrazine Simazine Ioxynil Bromoxynil Dinoseb	PSII isolated from *Synechococcus elongatus*	Amperometry	Diuron: LOD: 1 × 10^−9^ M, I_50_: 7 × 10^−8^ M Atrazine: LOD: 2 × 10^−9^ M, I_50_: 9 × 10^−8^ M Simazine: LOD: 4 × 10^−9^ M, I_50_: 2 × 10^−7^ M Ioxynil: LOD: 10^−7^ M Bromoxynil, Dinoseb: LOD: 10^−6^ M	[60]
Diuron Atrazine Simazine Terbuthyl-azine Diethylterbuthylazine	Thylakoid from *Spinacia oleracea* L., *Senecio vulgaris* and its mutant resistant to atrazine	Amperometry	LOD: 1.51 × 10^−8^ M−4.11 × 10^−8^ M I_50_: 1 × 10^−7^ M; for spinach thylakoids; detection in river water	[61]
Terbutryin	PSII-enriched thylakoid fractions from spinach	Colorimetry	LOD: 1.58 × 10^−7^ M	[62]
Atrazine Prometryne Terbuthyl-azine Diuron Linuron	Mutant strains of *Chlamydomonas reinhardtii* with engineered D1 protein	Fluorescence	S268C: LOD: 0.8 × 10^−11^ M–6.8 × 10^−10^ M IL: LOD: 1.0 × 10^−9^ M–3.0 × 10^−9^ M	[63]
Diuron	“BBY”-crude PSII preparation from spinach leaves	Amperometry	LOD: 1.1 × 10^−9^ M	[55]
Linuron Simazine	*C. reinhardtii* cells	Amperometry	Linuron: LOD: 6 × 10^−9^ M IC_50_: 1.2 × 10^−7^ M Simazine: LOD: 9 × 10^−8^ M, IC_50_: 2.3 × 10^−6^ M	[64]
Atrazine Bromacil Diuron	Thylakoids from spinach	Biosolar cell	Atrazine LOD: 0.37 μg L^−1^ Bromacil LOD: 0.21 μg L^−1^, Diuron LOD: 0.10 μg L^−1^; LR: up to ~15 μg L^−1^	[65]
Diuron Atrazine Ioxynil	Thylakoids from spinach	Amperometry	Diuron: LOD 1.3 ± 0.5 µg L^−1^, IC_50_: 2.1 µg L^−1^ Atrazine: LOD: 2.8 ± 0.3 µg L^−1^; IC_50_: 5.6 µg L^−1^ Ioxynil: LOD: 2.1 ± 0.3 µg L^−1^ IC_50_: 3.4 µg L^−1^; analysis of spiked water	[66]
Atrazine Prometryn Diuron	*C. reinhardtii* mutants	Fluorescence	LOD: 10^−10^ M for all mutants, Exception: F255N mutant is resistant to urea herbicides	[67]
Atrazine	Pure PS II cores and BBY particles from spinach	Amperometry	LOD: 1.15 × 10^−9^ M	[68]
Atrazine Isoproturon Diuron	PSII complex from *Synechococcus elongatus f. thermalis*	Amperometry	Atrazine: LOD: 6.4 × 10^−10^ M; IC_50_: 8.9 × 10^−7^ M Isoproturon: LOD : 5.5 × 10^−9^ M; IC_50_: 7.2 × 10^−7^ M Diuron: LOD: 4.6 10^−10^ M; IC_50_: 2.3 × 10^−7^ M	[69]
Diuron Linuron	Thylakoids from spinach	Amperometry	Diuron: LOD: 8.0 × 10^−9^ M; I_50_: 1.87 × 10^−7^ M; Linuron: LOD: 4.0 × 10^−9^ M; I_50_: 5.65 × 10^−8^ M	[70]
Atrazine Prometryn Diuron	Whole cells of Chlamydomonas reinhardtii	Fluorescence	Atrazine LOD: 5 × 10^−10^ M, Prometryn: 3.1 × 10^−10^ M Diuron: 4.81 × 10^−10^ M	[71]
Atrazine Propazine	*Chlorella pyrenoidosa* microalgae	Amperometry	Atrazine: LOD: 7 × 10^−7^ M; LR: 9 × 10^−7^ M–7.4 × 10^−5^ M Propazine: LOD: 4 × 10^−7^ M; LR: 6 × 10^−7^ M–1.2 × 10^−4^ M	[72]
Diuron	*Synechocystis* sp. PCC6803 cyanobacteria	Amperometry,	Diuron LOD: 5 × 10^−8^ M (cells in solution) and 5 × 10^−7^ M (immobilized cells)	[73]
Urea, diamine, triazine, phenols	Thylakoids from mutant spinach plants	Fluorescence	LOD: 3 × 10^−9^ M (in river water)	[74]
Diuron, Simazine Irgarol	*Chlorella mirabilis* algae	Fluorescence	Diuron: LOD: 0.067 µg L^−1^; LR: 0.50–25.0 µg L^−1^ Simazine: LOD: 0.705 µg L^−1^; LR: 1.00–50.0 µg L^−1^ Irgarol; LOD: 0.135 µg L^−1^ LR: 1.00–50.0 µg L^−1^; detection in seawater	[75]
Diuron	Thylakoids from spinach	Biosolar cell	I_50_: 67 ± 2 ng L^−1^	[76]

^1^ LOD: limit of detection; LR: linear range; I_50_: concentration of pesticide causing 50% inhibition.

**Table 2:** Different biosensors configurations for the detection of pesticides.

Pesticide	Detection Method	Limit of Detection	Linear Range	Reference
**Alkaline phosphatase**				
Metham-sodium	Fluorimetry	36.5 µM	75–480 µM	[7]
Tetradifon		4.1 µM	5–35 µM	
Fenitrothion		45.5 µM	135–270 µM	
2,4-dichlorophenoxyacetic acid	Amperometry	0.5 ppb	1.5–60.0 µg L^−1^	[70]
Malathion		0.1 ppb	0.2–45.0 µg L^−1^	
Paraoxon	Chemilumines-cence	50 ppb	n.d. *	[3]
Chlorpyrifos	Voltammetry	10^−9^ M	0.05–0.55 mM	[71]
**Organophosphate hydrolase**				
Paraoxon	Fluorescence	20 µM	up to 240 µM	[72]
**Tyrosinase**				
2,4-dichlorophenoxyacetic acid	Amperometry	0.6 ppt	0–10 ppt	[73]
Parathion	Amperometry	0.005 ppb	0.01–1 ppb	[74]
Carbaryl		0.008 ppb	0.01–10 ppb	
Atrazine	Amperometry	0.1 ppt	0.2 ppt–2 ppb	[82]
Atrazine	Amperometry	0.3 ppm	0.5–20 ppm	[83]
Atrazine	Amperometry	10 ppb	50 ppb–30 ppm	[84]
**Laccase**				
Methomyl	Square wave voltammetry	2.35 × 10^−7^ M	9.8 × 10^−7^–9.0 × 10^−6^ M	[75]
Carbofuran	Square wave voltammetry	0.022 mg/kg	4.98 × 10^−7^–5.88 × 10^−6^ M	[76]
Carbaryl		0.02 mg/kg	7.44 × 10^−8^–8.47 × 10^−7^ M	
Formetanate		0.21 mg/kg	2.49 × 10^−7^–7.46 × 10^−6^ M	
Pirimicarb		0.23 mg/kg	2.99 × 10^−7^–5.66 × 10^−6^ M	
Ziram		0.02 mg/kg	2.49 × 10^−7^–5.66 × 10^−6^ M	
Pirimicarb	Square wave voltammetry	1.8 × 10^−7^ M	9.95 × 10^−7^–1.15 × 10^−5^ M	[77]
Formetanate	Square wave voltammetry	95 nM	n.d. *	[85]
**Heme-containing enzymes**				
Aldrin	Amperometry	8 × 10^−6^ M	9.08 × 10^−6^–4.54 × 10^−5^ M	[78]
Heptachlor		n.d. *	8.91 × 10^−6^–4.46 × 10^−5^ M	
Glyphosate	SWV	30 µg L^−1^	0.1–4.5 mg L^−1^	[86]
Aminomethylphosphonic acid	Amperometry	1.µg L^−1^	1.5–7.5 mg L^−1^	[87]
Glyphosate		0.16 μg L^−1^	2.0–14.0 µg L^−1^	
Dichlofenthion	Amperometry	24 µM	5–100 µM	[88]
**Urease**				
Atrazine	Enzyme Field Effect Capacitive System	0.12 µM	0.1 µM–10 mM	[89]
Glyphosate	Potentiometric	0.5 ppm	0.5 ppm–50 ppm	[79]

* n.d. not determined.
